# The anatomy of empathy: Vicarious experience and disorders of social cognition

**DOI:** 10.1016/j.bbr.2016.05.048

**Published:** 2016-09-15

**Authors:** Patricia L. Lockwood

**Affiliations:** Department of Experimental Psychology, University of Oxford, Oxford, United Kingdom

**Keywords:** Empathy, Pain, Reward, Anterior cingulate cortex, Anterior insula, Psychopathy, Autism

## Abstract

Empathy, the ability to vicariously experience and to understand the affect of other people, is fundamental for successful social-cognitive ability and behaviour. Empathy is thought to be a critical facilitator of prosocial behaviour and is disrupted in a number of psychiatric and neurological disorders. Research has begun to uncover the neural basis of such ‘vicarious experience’, which has been studied as a proxy measure of empathy. Together, these studies have identified portions of the insula and anterior cingulate cortex as critically involved. A key debate is whether overlapping or non-overlapping brain areas respond to personal and vicarious experience. This review will highlight emerging evidence for both types of brain response. Importantly, animal models have suggested that there are central divisions between the anterior cingulate gyrus and anterior cingulate sulcus that may be crucial for understanding social behaviour. Attention to this specific anatomy of vicarious processing could therefore help shed light on the functional profile of empathy. Studies in individuals with psychopathy and autism spectrum disorders have found that vicarious experience is atypical. However, the precise nature of these atypicalities is mixed. Understanding the mechanisms of vicarious experience can enhance our knowledge of the neural basis of empathy and, ultimately, help those with disorders of social cognition and behaviour.

## Highlights

• The anatomy of vicarious experience in animal and human studies is reviewed.
• The ACC gyrus and anterior insula are central to vicarious experience.
• Vicarious experience can rely on both shared and non-shared neural responses.
• Aspects of vicarious experience may be atypical in psychopathy and autism.

## 1 Introduction

Humans are highly social creatures, living in complex social environments and spending much of their lives interacting with, and thinking about, others. During social interactions, a crucial first step is to perceive events that will have an impact on others. Processing these events is key for empathising and successful social interaction. This includes resonating with others’ pain, but also feeling the joy of other people. Studies in the field of social neuroscience have attempted to identify the neural substrates of such ‘vicarious experience’. In human studies, overlapping neural responses to events for self and others have often been interpreted as a proxy measure of empathy [1], [2].

Empathy is thought to be an important motivating factor for prosocial behaviour [1], [3], [4], [5], [6] and is altered in a number of psychiatric and neurological disorders including psychopathy and autism [7], [8]. Understanding the mechanisms of empathy is therefore not only of scientific interest but, in the long term, could have practical implications for promoting prosocial interactions and helping individuals with disorders of social behaviour.

In this review, the background, definitions and structure of empathy will be addressed. Studies that have examined the neural basis of empathic/vicarious experience will be reviewed and it will be shown that findings support both overlapping and distinct neural responses to personal and vicarious experience. In particular, subdivisions in anterior cingulate cortex and insula are suggestive of relative specificity, as well as overlap, when processing information about others. Finally, the possible implications of the extant evidence base for understanding disorders of social cognition and future directions are critically discussed.

### 1.1 What is empathy?

The psychologist Edward Titchener first introduced the word “empathy” into the English language over 100 years ago, as a translation of the German word *Einfühlung* (“feeling into”). Whilst there is no complete consensus as to the precise definition of empathy, most theorists agree that empathy is, broadly, the ability to vicariously experience and to understand the affect of other people [1], [6], [9], [10], [11], but see [12] for a different perspective.

An important distinction within the structure of empathy is often made between emotional/affective and cognitive aspects. Affective empathy is commonly understood as an affective state (such as the experience of emotion, pain or reward), caused by sharing the state of another person through observation or imagination of their experience [1], [5]. Although an observer’s emotional state is isomorphic with that of another person, the observer is aware that someone else is the source of that state [5]. Cognitive aspects of empathy are commonly referred to as perspective taking, mentalising or theory of mind. Combined, these processes enable an observer to understand another person’s beliefs, desires and emotions [13]. In this review, both components are seen as important contributors to the experience of empathy (in line with [9]). However, it is important to note that some authors define empathy as comprised only of the “affective” components and label the “cognitive” components as a separate but related construct of “theory of mind” or “mentalising” on the basis that they rely on largely distinct neurocognitive circuits (e.g. [14]).

It is generally agreed that affective empathy should be distinguished from emotion contagion, mimicry, empathic concern, compassion and sympathy [1], [9]. Although these processes usually occur in similar contexts they have been distinguished from empathy conceptually. For example, a recent model of empathy, entitled the self-to-other model of empathy (SOME; [9]) highlights that emotional contagion is a key precursor to empathy but does not have to involve a distinction between self and other. Thus, although emotion contagion may be necessary for empathy, and is an instance of a vicarious experience, on its own it is not sufficient due to a lack of self-other distinction. Empathic concern, which is also called ‘sympathy’ or ‘compassion,’ involves ‘feeling for’ the other person [1] and is associated with motivation to alleviate their suffering. Empathic concern is frequently equated with empathy. However, because empathic concern does not necessarily involve any vicarious experience, it is distinguishable from affective empathy.

Various self-report and behavioural measures have been developed to capture variability in empathy. One of the first of these measures, the Interpersonal Reactivity Index (IRI, [15]) has been hugely influential in the field of empathy research. The IRI contains subscales measuring empathic concern, perspective taking, personal distress and fantasy. The perspective taking and fantasy subscales are suggested to measure cognitive empathy, whereas the empathic concern and personal distress subscales are thought to assess affective empathy. However, it is unclear how the different components of the IRI relate to empathy as defined in this review and the field more generally. For example, the fantasy scale contains items such as “I daydream and fantasize, with some regularity, about things that might happen to me” which does not measure feeling or understanding the affect state of another person. The personal distress subscale asks questions about personal responses to emergency situations e.g. “When I see someone who badly needs help in an emergency, I go to pieces.” and such responses may involve both empathising and sympathising [16]. Moreover, the IRI possesses no specific measure of vicarious experience, only empathic concern (sympathy), and thus does not measure the conceptualisation of empathy adopted in the current review and in the field more generally (e.g [1], [6], [9], [10]).

To overcome these limitations and to create an instrument that assesses the multidimensional nature of empathy more closely and reflects current definitions of empathy, the Questionnaire of Cognitive and Affective Empathy (QCAE) was developed by Reniers and colleagues [17]. The QCAE is an instrument devised to measure five key components of empathy. In the development of the QCAE, two raters selected items from other commonly used empathy measures (e.g. Hogan Empathy Scale (HES;[18])), Interpersonal Reactivity Index (IRI; Davis, 1983), Balanced Emotional Empathy Scale (BEES;[19]), and Empathy Quotient (EQ; [20]) if they were deemed to measure empathy (see items below). Items deemed to measure other processes (e.g. sympathy) were not included. These items were then subjected to an exploratory factor analysis to identify the underlying structure of their associations and then to a confirmatory factor analysis in a separate sample to confirm the identified structure, which is the ‘gold standard’ approach to questionnaire development and validation [21].

The five subscales identified by this procedure were: perspective taking (e.g. “I can easily tell if someone else wants to enter a conversation.”); online simulation (e.g. “Before criticizing somebody, I try to imagine how I would feel if I was in their place.”); emotion contagion (e.g. “I am happy when I am with a cheerful group and sad when the others are glum.”); peripheral responsivity (e.g. “I often get deeply involved with the feelings of a character in a film, play, or novel.”); and proximal responsivity (e.g. “I often get emotionally involved with my friends’ problems”). These subscales can be further grouped into two factors that the authors named cognitive and affective empathy. Cognitive empathy comprises the subscales of perspective-taking and online simulation, whereas affective empathy comprises the subscales of emotion contagion, peripheral responsivity and proximal responsivity. The QCAE has been shown to have well-validated psychometric properties [17]. and measures empathy as a multidimensional phenomenon comprised of related but separable constructs.

In neuroimaging studies the majority of work has focused on studying neural responses to vicarious experience (events that will have an impact upon others including others’ pain and reward) as a proxy measure of empathy, and examining variability in these neural responses as a function of self-reported empathy. Although many proxy behavioural measures of empathy have been developed (e.g. Reading the Mind in The Eyes, Multifaceted Empathy Test [22], Animations Test [23]) this review will focus on neural responses during vicarious experience, particularly the perception of others’ pain or reward, in order to draw parallels across studies more closely, and also with studies in non-human animals.

## 2 Anatomy of the anterior cingulate cortex and anterior insula

**Fig. 1:**
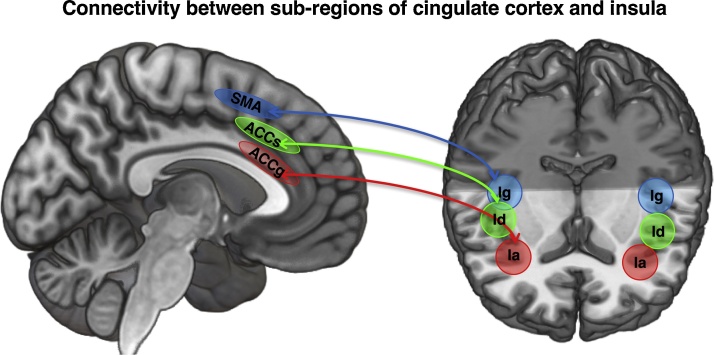
Connectivity between cytoarchiectonic sub-regions of the cingulate cortex and insula. SMA = supplemental motor area, ACCs = sulcal portion of the anterior cingulate cortex, ACCg = gyral portion of the anterior cingulate cortex, Ia = agranular anterior insula, Id = dysgranular mid insula, Ig = granular posterior insula.

The anterior cingulate cortex (ACC) and anterior insula (AI) are key brain regions that respond during vicarious experience [2], [24], [25], [26]. Consequently, understanding the functional anatomy of these regions is crucial to understand how vicarious information is processed and to enable comparisons to be drawn across species [27] (Fig. 1).

**Fig. 2:**
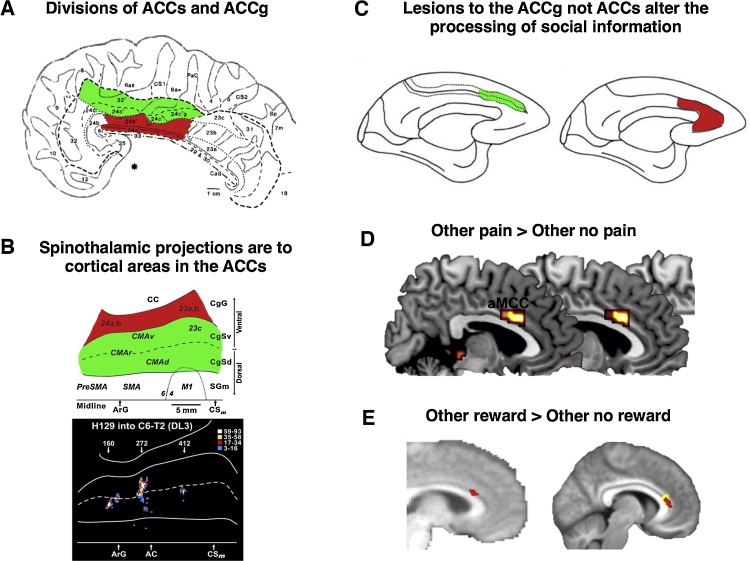
Divisions of the cingulate cortex and relevance for the processing of vicarious experience (**A**) Cytoarchitecture of the ACC adapted from Vogt et al. (1995). The areas shaded in green lie in the anterior cingulate sulcus (ACCs) The areas shaded in red lie in the anterior cingulate gyrus (ACCg). Extant evidence suggests the processing of social information is primarily in the gyral portion of ACC (areas 24a’ and 24b’) and extend on average 22 mm posterior and 30 mm anterior the anterior commisure. (**B**) spinothalamic projections (the major pathway for nociceptive and thermal information) are found in the cingulate sulcus and not the cingulate gyrus. These projections code for “pain” as well as motor control. Figure adapted from Dum et al., 2009 (**C**) Lesion site of the ACCs (green) and ACCg (red) adapted from Rudebeck et al. (2006). The lesions that affected the gyrus, but not the sulcus, caused disruptions to social behaviour and disrupted the processing of social stimuli. (**D**) Responses in the ACCg to others pain compared to no pain (labelled aMCC in the figure), taken from a *meta*-analysis of vicarious pain studies (Lamm et al., 2011). (E-left) Responses of the ACCg to the net-value (cost-benefit) of anothers’ predicted reward compared to no reward (red) taken from Apps & Ramnani, 2014. (E-right). Responses of the ACCg to another person’s likely compared to unlikely reward (red), adapted from Lockwood et al. (2015). (For interpretation of the references to colour in this figure legend, the reader is referred to the web version of this article.)

The cingulate cortex is anatomically and functionally heterogeneous and consists of distinct cytoarchitectonic zones (i.e. differences in cellular structures indicative of functional subdivision) (See Fig. 2a) [28], [29]. These zones have been broadly labelled as retrosplenial, posterior, mid (MCC) and anterior (ACC) [28], [29], [30], [31]. In both the MCC and ACC there are further subdivisions between the sulcus (MCCs/ACCs, henceforth ACCs) and the gyrus (MCCg/ACCg, henceforth ACCg) that point to distinct functional properties (Fig. 2). The ACCs (often referred to as ‘dorsal ACC, regions 24c/c’ and 32/32′) has connections to primary motor, premotor, supplementary motor (SMA) and pre-supplementary motor (pre-SMA) cortices intraparietal sulcus, orbitofrontal cortex and nucleus accumbens [32], [33], [34]. Posterior portions of the ACCs are often considered motor aresa based on their direct projections to the spinal cord [35], [36], [37] and electrical stimulation of neurons in ACCs results in limb movements [38]. In contrast, the ACCg (areas 24 a, b, a’ and b’) has connections to posterior portions of the superior temporal sulcus, the temporo parietal junction and dorsomedial prefrontal cortex (dmPFC) [39], [40], [41], [42] that are well known to be engaged when processing the mental states of others [13]. Importantly, the ACCg has strong connections to anterior, but not posterior insula [43]. Both ACCs and ACCg have common connections to medial and lateral portions of the orbitofrontal cortex [44], [45] and to the nucleus accumbens [46], [47] suggesting involvement of both regions in processing rewards. However, there is no evidence of connections from TPJ, pSTS and dmPFC to ACCs.

**Fig. 3:**
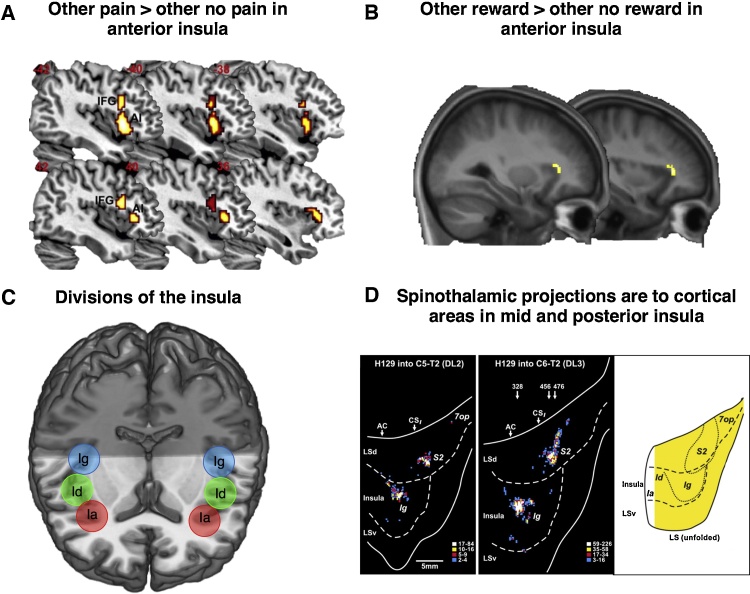
Divisions of the insula cortex and relevance for the processing of vicarious experience **(A)** Responses in the anterior insula to others pain compared to no pain, taken from a *meta*-analysis of vicarious pain studies (Figure adapted from Lamm et al., 2011). **(B)** Responses of the anterior insula to others likely compared to unlikely reward, from a reanalysis of data in Lockwood et al., 2015. Peaks survive small volume correction for a bilateral anterior insula structural mask. **(C)** Subdivisions of the insula based on cytoarchitecture, Ia = agranular portion of the anterior insula, Id = dysgranular portion of the mid insula, Ig = granular portion of the posterior insula. Figure adapted from Dum et al., 2009. **(D)** spinothalamic projections (the major pathway for nociceptive and thermal information) are found in the mid and posterior portions of the insula (Ig, Id) and not the anterior insula (Ia). These projections code for “pain” as well as motor control.

The insula is also an anatomically and functionally heterogeneous region. Based on the degree of granularity, modern descriptions of the insula generally agree on three subdivisions [43]. These are anterior agranular cortex (anterior insula), a middle dysgranular cortex (middle insula) and a posterior granular cortex (posterior insula)[43] (Figs. 1 and 3 c). In addition to differences in cytoarchitecture, these subregions feature distinct connectivity patterns in both human and non-human primates [43], [48], [49]. The AI has connections to the ACCg, frontal operculum, orbitofrontal cortex, dorsal and ventral temporal pole, and sensory areas such as the somatosensory and opercular areas of the parietal lobe [43], [48], [49]. The middle insula has connections to the ACCs, frontal operculum, VMPFC, orbitofrontal cortex, to the secondary somatosensory area, to the superior temporal sulcus, ventral striatum, and amygdala [43], [48], [49]. The posterior insula is connected to the SMA, VMPFC, temporal poles, secondary somatosensory area, and dorsolateral striatum [43], [48], [49]. The posterior insula receives projections from the spinothalamic pathway, the major pathway for processing nociceptive information, whereas these projections do not seem to reach AI(Fig. 3D) [50]. Stimulation of neurons in the posterior insula but not other portions elicits feelings of pain and warmth [49]. Importantly, the AI connects to ACCg whereas the mid and posterior insula are primarily connected to ACCs and SMA respectively (see Fig. 1).

Overall, the anatomical and functional profile of ACCg and AI suggest that these regions may be involved in processing social information, that is, information that is directed to or about other people.

## 3 Animal studies of vicarious experience

Research in non-human animals can help us to identify neural regions that may be involved in empathising, as it allows us to directly record the activity of neurons during vicarious experience, and to cause focal lesions (although of course we cannot infer from these studies that animals are experiencing the phenomenon of empathy). The majority of this work has focused on the observation of others rewards rather than pain and on the ACC rather than insula.

As mentioned in the previous section, there is converging evidence that the ACCg plays a key role in social cognition and behaviour in both human and non-human primates [26], [51], [52], [53], [54], [55], [56], [57], [58], [59], [60]. In particular, animal models have suggested that there are important divisions between the ACCg and the ACCs that are crucial for understanding social behaviour [57], [59]. It has been argued that the ACCg and ACCs both processes information that conforms to the principles of reinforcement learning theory but the ACCg does this in social contexts whilst the ACCs does so in ‘non-social’ contexts [54] (Fig. 2).

Lesions to the ACCg impair the processing of social stimuli and cause a reduction in the execution of social behaviours, whereas lesions to the ACCs and orbitofrontal cortex do not [59](Fig. 2C). A seminal study by Chang and colleagues was the first to record from neurons in the ACCs, ACCg and OFC during a social decision-making task [57]. In their task monkeys were assigned roles of actor (self) and recipient (other). On 50% of trials the actor chose between cues that delivered rewards to themselves, the other monkey or to neither. On the other 50% of trials the computer made the choices for them. They observed that a greater proportion of neurons in the ACCg, compared to the ACCs and OFC, responded to cues that predicted rewards for other monkeys and also to decisions to allocate rewards to other monkeys. This is rather striking, because to date very few studies have found neurons that process specifically “other” related information. Sallet et al. [60] reported further evidence for a key role of the ACCg in social behaviour. They found that non-human primates with larger social networks had increased grey matter volume in the ACCg compared to those with smaller social networks [60]. Taken together these studies provide support for the claim that the ACCg is important for processing vicarious information, and also in social behaviour more widely. This profile of responses to information about other agents does not seem to be the same for the ACCs.

## 4 Human studies of vicarious experience

In human studies of vicarious experience, paradigms have been used that require a participant to observe another individual in pain (for a *meta*-analysis see [24]) others’ emotional facial expressions [61], [62], pleasant affect [63], [64] and reward [26], [52], [65], [66]. The following sections will focus on studies of observing others’ pain and reward as key aspects of vicarious experience and because these processes have been investigated in humans the most extensively. It will be shown that such processing of others pain and reward may rely on both “shared” and “non-shared” neural regions.

### 4.1 Vicarious pain

Many studies investigating the neural basis of vicarious experience have focused on the observation of other people in pain as a proxy measure of empathy. Pain can be broadly defined as the perception of actual or threatened tissue damage and the private experience of unpleasantness, e.g. that it ‘hurts’. Areas of the brain involved in the first-hand experience of pain include the ACC (mainly ACCs), insula (mainly posterior and mid portions), thalamus, brain stem and periaqueductal gray [67], [68], [69], [70]. Converging evidence in humans suggests that the primary and secondary somatosensory cortices encode information related to sensory features of pain such as location and intensity [69], [71], [72], [73], whereas the ACC and insula are related to affective and motivational aspects of pain experience [67], [69], [70], [74]. This work has therefore provided the basis to investigate neural response to other’s pain, and to examine whether the same regions that process personal pain also respond to vicarious pain.

One of the first studies to investigate the neural responses to the observation of other people’s pain, as a proxy measure of empathy, was conducted by Singer and colleagues [75]. In their seminal study, participants experienced a painful stimulus whilst undergoing functional magnetic resonance imaging (fMRI). On “self” trials this painful stimulus was delivered to themselves, but on “other” trials participants observed cues that signalled that their partner, who was present in the same room, was receiving a painful stimulus. Singer et al. [75] found that the AI and ACCs responded both when the participants themselves received the painful stimulus and when they viewed a cue that indicated that their partner received a painful stimulus. In contrast, response in the secondary somatosensory cortex and primary somatosensory cortex was associated with greater response to the pain participants received themselves, compared to pain received by their partners. Trait empathic concern, as measured by the Interpersonal Reactivity Index [15] and the Balanced Emotional Empathy subscale [19], were positively associated with blood oxygen level (BOLD) responses to others pain (compared to no pain). Taken together, these findings supported the idea that the observation of others experiences activates similar neural regions to one’s own experiences, which was interpreted as a neural marker of empathy.

Since this seminal study, many studies have found AI, ACCs and ACCg responses to the observation of others’ pain (Fig. 2, Fig. 3) (for a *meta*-analysis see [24]). One distinction that has been drawn is between studies that have used cue-based paradigms (e.g. [75], [76], [77], [78]) and studies that have used picture based paradigms (e.g. [79], [80], [81]). In cue based paradigms participants view cues that indicate that pain will be delivered to them or another individual who is not in an fMRI scanner. In contrast, picture based paradigms involve viewing body parts such as hands and feet either in situations likely to cause pain or likely not to cause pain. In a recent *meta*-analysis it was found that only picture-based paradigms elicited responses in somatosensory cortices [24]. The authors argued that this suggests that activation of somatosensory cortices during vicarious pain paradigms may be related more to the perception of movement and touch rather than vicarious experience per se.

### 4.2 Vicarious reward

A more recent development in research on vicarious experience has been the investigation of neural responses to others’ rewards [52], [65], [66], [82]. Here, vicarious reward is defined as the perception of another person’s anticipated or consumed reward. It is important to note that the majority of studies that have investigated brain responses to self and other pain have focused on the technique of conjunction analysis (self pain > no pain ∩ other pain > no pain). Studies examining others’ reward have focused on interaction effects to identify neural responses exclusively to vicarious reward (other reward > no reward + self no reward > reward).

It is well known that rewards are a powerful motivator of human and animal behaviour. The neural regions that are involved in reward processing are becoming increasingly well understood [83]. This includes the ACCg, ACCs VMPFC, amygdala and striatum [83], [84], [85]. In social environments, it is also important to process information about rewards predicted for and delivered to others in order to effectively cooperate or compete or empathise with them [86], [87].

In one early study Mobbs et al. [66] asked participants to watch videos of two game show contestants answering questions about their political and social views in a way that was socially desirable (SD) or socially undesirable (SU). Participants were then scanned whilst they watched these two players (SD and SU) play a card-guessing game where they could win or lose money. Afterwards, the participants played the game themselves. Mobbs et al. [66] found that activity in the ventral striatum, subgenual anterior cingulate cortex (sgACC) and VMPFC correlated with the difference between watching the SD and SU contestant win. Only the ventral striatum response was also observed when the participants played themselves. These findings suggest both common and distinct neural regions may be involved in vicarious experience, since many of these regions (except the ventral striatum) responded to viewing rewards for other people and not for oneself. One potential limitation was that Mobbs and colleagues used a block design so were unable to separate neural responses to reward prediction and reward consummation, which in the domain of first-hand reward experience may be somewhat distinguishable [88]. It could be that these regions only responded to rewarding *outcomes* for others and that separate regions signal the *anticipation* of rewards for others. Indeed, there is evidence that the striatum may not show overlap between processing of personal and vicarious reward when reward cues and outcomes are separated or in other studies of vicarious reward [52], [89].

Apps et al., [52] examined brain activity at the time of cues that signalled the net-value (benefit-cost) of anticipated rewards (benefit) and the level of effort (cost) to be incurred either by a participant themselves or when monitoring the net-value for another participant [52]. They observed that the ACCg specifically signalled the net-value of rewards to be gained by others, but did not respond to rewards to be gained by oneself. In contrast, activity in the ventral striatum signalled the net-value only for the participants themselves. This suggests that the ACCg may play a specific role in vicarious reward, whereas the ventral striatum may be more involved in processing first-person reward (see also [89]).

Further support for a key role of the ACCg in vicarious reward comes from a recent study by Lockwood et al. [26]. In their study participants viewed cues that predicted a high or low probability of reward either for themselves or a confederate participant seated outside of the scanner. They found not only that the ACCg signalled the likelihood of a reward being delivered to another, but also that ACCg response significantly covaried with trait emotion contagion (a necessary condition for empathy [9]). In individuals high in emotion contagion, the ACCg was specialised for processing others’ rewards exclusively, but for those low in emotion contagion, this region also responded to information about the subject’s own rewards. This suggests that empathy can modulate the extent to which self compared to other information is processed, not only the extent to which social information is encoded in ACCg.

A reanalysis of the Lockwood et al., [26] data also found activation in bilateral AI for the contrast other high probability > other low probability of reward, suggesting responses in AI to others reward (small-volume corrected with anatomical mask of AI, Fig. 3B). This mirrors findings of responses to others’ pain (see Fig. 2, Fig. 3). Other neuroimaging studies have also shown ACCg and AI (uncorrected) responses to the observation of unexpected rewards for others [51] and when another person’s predictions about rewards are incorrect [53]. A recent *meta*-analysis of studies of rewarding outcomes in social contexts identified common and distinct neural regions that respond to personal and vicarious reward [89]. Whereas personal reward was encoded to a greater extent in the ventral striatum, vicarious reward was encoded to a greater extent in the dorsomedial prefrontal cortex and posterior superior temporal sulcus. However, this *meta*-analysis included both studies of monetary and social rewards (such as positive social feedback) and there is still much debate as to whether there is a ‘common-currency’ of neural regions responding to all types of reward (e.g. money, food, social stimuli) or whether these different types of reward are processed in distinct neural regions [87]. This *meta*-analysis does importantly however identify ‘non-shared’ neural responses to personal and vicarious reward.

### 4.3 Summary

Taken together, the neural processing of other people’s experiences of pain and reward primarily recruits the AI, ACCs and ACCg. Responses have also been observed in the inferior frontal gyrus(IFG), sgACC, vmPFC and dmPFC and perhaps ventral striatum. Studies of vicarious pain have suggested that there is overlap in the processing of personal and vicarious pain whilst studies of vicarious reward have suggested that there are regions that specifically respond to vicarious reward and not to personal reward.

## 5 Does empathy rely on shared representations?

Whether empathy relies on overlapping or non-overlapping processing of personal and vicarious experience is still widely debated (see [90], [91], [92] for recent discussions). Historically, this debate can be seen as stemming from theoretical disagreements as to the nature of social processing. Theory-theorists argued that we understand the minds of others by forming a folk psychological theory about another’s mental states beliefs and desires [13] whereas simulation theorists argue that we understand the minds of others by a directly (or indirectly) simulating their mental states [93], [94]. Whereas theory–theory is consistent with identifying brain areas that specifically respond to vicarious experience, simulation theory posits that neural activations should be overlapping in order to be correlates of empathy. However, at both the neural as well as psychological level these theoretical perspectives are not necessarily mutually exclusive. Whilst it is clear that many areas of the brain respond to both first-person and third-person experience [24], [25], [89] there is also evidence that there is some specificity when functional anatomy is considered [26], [51], [52], [53], [54], [55], [56], [57], [59]. Moreover, particularly in the domain of positive affect, there are many cases where vicarious experience does not depend on a shared-representation [26], [54], [89], [95].

Support of shared representations account is evidenced by studies showing areas of overlap to first-person and third person pain [24]. Moreover, a recent study exploited the well-known placebo analgesia effect whereby individuals report pain reduction after being instructed they are being administered a potent painkiller, which is actually an inactive compound (e.g. [96]) to investigate shared representations. Rütgen and colleagues found that both self report measures of empathy and neural responses to others pain (in AI and ACCs) were affected by placebo analgesia [97], suggestive of shared functional overlap between self and simulating other pain.

However, there is also support of non-shared representations being involved in vicarious experience. Patients who have a congenital insensitivity to pain activate ACC (ACCg although cluster extended over ACCs) and AI when seeing others suffering pain, even they do not experience pain themselves [98]. Studies have also found activation in ACCs and AI when people watch medical procedures such as pinpricks, which appear aversive but do not actually cause any pain, speaking against a strong shared representations account of vicarious experience [99], [100]. In studies of neural responses to others’ reward, the ACCg, sgACC and VMPFC respond specifically to rewards delivered to others and do not show overlap with the processing of information for oneself [26], [51], [52], [53], [66], [82]. In one of these studies, response in the ACCg varied as a function of emotion contagion with those lowest in emotion contagion processing both self and other information in ACCg, but in those high in emotion contagion this region was specialised only for processing rewards for others. As outlined above, animal studies have suggested that the ACCg is central to social behaviour and that this region does not seem to be involved in coding rewards for oneself [59]. Moreover, Chang et al. [57] identified neurons in the ACCs that responded when to no-one receiving a reward and Apps et al. identified signals in ACCs that responded to unexpected rewards for others but also for a computer [51]. The ACCs is the cingulate sub-region where many fMRI activations to vicarious and personal pain have been observed. This suggests that some aspects of shared representations may not reflect vicarious experience per se but a domain-general coding of information.

As mentioned previously, an aspect that is often overlooked is the specific anatomy of vicarious experience. Although subdivisions of the insula have been referred to (e.g. [101]) subdivisions of the cingulate cortex are not often considered. Anatomically, there may be relative specificity for processing aspects of vicarious pain and reward (responses in AI and ACCg, see Fig. 2, Fig. 3) compared to self pain and reward (responses ACCs and posterior insula (for pain)). An important study that examined spinothalamic (the major pathway for transmitting nociceptive and thermoceptive information to the cerebral cortex) projections to cortex revealed that these projections were mainly to the mid and posterior portions of the insula, secondary somatosensory cortex (Fig. 3c) and the ACCs (Fig. 2b) [50], [102], [103]. This suggests that personal pain may involve the ACCs and posterior insula, not ACCg and AI (Fig. 2B).

The involvement of both shared and non-shared representations in vicarious experience is perhaps not surprising, given the different sensory inputs during personal and vicarious experience. At the same time, there are common processes that contribute to personal and vicarious experience. The ACCs is often considered a motor area that codes signals of value, motivation, pain and foregone rewards [50], [103], [104], [105]. An important aspect of personal and vicarious experience is guiding of our subsequent actions, which these signals may motivate. Understanding the functional anatomy of brain regions involved in vicarious experience may therefore be crucial when moving forward with understanding how such information is processed in the brain. Consideration of anatomy can also help to delineate what aspects of vicarious experience might be shared and which are non-shared.

## 6 Disorders of vicarious experience

### 6.1 Psychopathy

Psychopathy is a disorder characterised by a constellation of cognitive and behavioural atypicalities including callousness, shallow affect, lack of guilt, antisocial behaviour and impulsivity (e.g. [106], [107], [108], [109], [110]). These individuals commit a disproportionate amount of violent crime, and place a substantial economic and emotional burden on society [7]. The ability of individuals with psychopathy to seriously violate the rights of others’ is thought to highlight a disturbance in an appropriate empathic/vicarious response to other people [106], [107], thus psychopathy is perhaps the archetypal empathy disorder.

In children, there is abundant evidence that psychopathic traits and behaviours can be observed and that the behavioural and affective disturbances that are seen generally mirror those observed in adults with high levels of psychopathic traits [107], [111], [112]. In childhood, high levels of antisocial behaviour can be diagnosed as conduct disorder (DSM-5). Particular subsets of children with conduct disorder can also have elevated levels of psychopathic traits, which are termed callous-unemotional (CU) traits in research studies and “limited prosocial emotions” in the new DSM-5 guidelines. In the following section, studies from these different populations will be discussed, together with the assumption that they can all contribute to informing us about the profile of psychopathy.

There is a wealth of evidence that psychopathy may be associated with atypical vicarious experience. Behavioural evidence suggests that adults and children with psychopathy show reduced physiological responses to the distress of others [113], blunted emotional reactivity to aversive stimuli [114], impaired recognition of and reactivity to distress [107], [115] and positive facial expressions in others (e.g [115], [116]). Similarly, adults with high levels of psychopathic traits seem to show reduced ratings of affective resonance to other people’s positive and negative emotions [117], [118], [119]. They also report less enjoyment of interacting prosocially with others, suggesting that they may have reduced vicarious experience more broadly [120]. Children with conduct disorder show behavioural impairments in tasks related to affective empathy but not in tasks probing processes related to cognitive empathy [111], [112].

Similarly, neuroimaging studies have also found evidence of atypical vicarious experience including atypical neural responses to the perception of others’ pain and emotional experiences (to date, the perception of rewards for others has not been examined). For example, male offenders with high levels of psychopathy have been found to show reduced responses in IFG and ACCg, but greater responses in the insula when observing facial expressions of others’ in pain [121]. Other studies have found that when observing videos depicting hands in emotional situations such as being caressed or hit by another hand, incarcerated males with psychopathy have lower activation in AI, IFG, a posterior portion of the ACCg, and amygdala [122]. However, when instructed to “empathise” with the actors’ hands differences in neural responses between the groups were reduced. In subclinical samples of high psychopathic traits, reduced responses have been found in the amygdala and AI when viewing other people’s emotional facial expressions (e.g. [61]) and with variability in neural responses to the perception of other people’s pain in a posterior portion of the ACC (mask covering the ACCg and ACCs), AIand IFG [123].

fMRI studies in adolescents with conduct problems have also found atypical neural response to other people’s distress or pain compared to controls [124], [125], [126], [127], [128], [129]. For example, Lockwood et al. [125]. measured BOLD responses to images of other people in pain in children with conduct problems and varying levels of CU traits compared to a control group of children without conduct problems or CU traits. They found that across the groups children with conduct problems showed reduced neural responses to the images of others in pain in ACC (a structural mask was used but peak activation was in ACCg), AI and IFG. Crucially, those children that were rated to be the most callous by their parents and teachers showed the lowest neural responses to others pain in AI and ACCg [125]. Taken together these findings suggest that individuals with psychopathy/psychopathic traits have atypical vicarious experience and these atypical responses are reflected in similar regions to vicarious experience in healthy people.

### 6.2 Autism spectrum disorders

Autism spectrum disorders (ASD) refer to a class of developmental disorders characterised by impaired social and communication skills and a restricted repertoire of interests and activities. Several decades of research indicate that individuals with ASD have difficulties with “cognitive” aspects of empathy (see [130]). ASD has often been described as a disorder associated with “poor empathy” [131]. However, it is important to note that the nature of their social information processing deficits and behaviours seem very different from those seen in individuals with psychopathy/psychopathic traits [9], [111], [112], [119], [132].

A number of studies measuring cognitive and affective processes related to empathy have found impairments in cognitive perspective-taking but not empathic concern in adults [22] and reduced cognitive perspective-taking (cognitive empathy) but not affective resonance/affective empathy in children with ASD [111], [112]. Studies focusing solely on affective processing have found evidence of preserved affective processing, including normal skin conductance response to others’ negative emotions when emotions are unambiguous and presented under conditions of low distraction [133]. Some theorists have argued that affective empathy is actually heightened in individuals with ASD [134] and this is consistent with reports of greater empathic facial affect in children with ASD compared to controls [135].

However, individuals with ASD have also been found to have lower scores on the empathy quotient, a self-report questionnaire of empathy, compared to typically developing individuals [20]. Another study found that parents of children with ASD reported their children to be less concerned about emotional situations and less responsive to distress cues than control children [136]. Nevertheless, it is unclear in studies that do find affective empathy impairments whether these relate to problems in social responsivity rather than affective empathy per se.

Neuroimaging studies of vicarious experience in ASD have largely focused on the perception of others pain [77], [137], [138], [139]. Studies that have examined neural responses to others’ reward have done so only in the context of iterative games such as the trust game [140] and shown atypical responses in the ACC (see also [141] and [142] for evidence of atypical ACC responses and connectivity in ASD). However, iterative games do not provide a ‘pure’ measure of vicarious experience, as these tasks do not clearly separate out self and other, or the decision about how to respond to the other from the perception of the other, and therefore a detailed discussion of these tasks is beyond the scope of this review.

Within the domain of vicarious pain, findings from studies are somewhat mixed with evidence for normal, increased and decreased brain responses. For example, Bird and colleagues found no group differences to the perception of others pain in individuals with ASD and controls matched for levels of alexithymia (a sub-clinical condition defined by an inability to identify and describe one’s own feelings that is highly comorbid with autism) [77]. However, they found that the degree of alexithymia was associated with lower responses to others’ pain in AI in both groups. This suggests that it is not autism per se but high levels of alexithymia that may be associated with reduced vicarious responses. Similarly, Hadjikhani et al. [138] found no group differences to the observation of facial expressions of pain in a relatively large sample of individuals with ASD (n = 38) and a control group (n = 35) also suggesting similar vicarious experience in ASD and controls (differences in medial prefrontal cortex were seen at a reduced statistical threshold). In contrast however, another fMRI study found that individuals with ASD had reduced neural responses in AI and ACC (potentially covering ACCg and ACCs) when they viewed other people in pain, but there were no significant moderating effects of alexithymia [139]. One explanation for these different findings is that the variance in alexithymia scores was more restricted in the latter study [139] compared to Bird et al. [77] (SD 3.8 in [139] vs. 11.8 in ([77])).

Gu et al. [137] compared skin conductance responses (SCR), behavioural responses of pain discriminability, fMRI and dynamic causal modelling in individuals with ASD and controls during a picture-based perception of others’ pain paradigm. Interestingly, they found that compared to controls adults with ASD showed *increased* SCR responses when viewing images of other people in pain as well as *increased* neural responses in the AI. However, individuals with ASD showed reduced discriminability behaviourally when asked to judge whether other people in photographs were experiencing pain or not.

Finally, although most studies have focused on processing others’ pain, a handful of studies have examined neural responses to processing social information more broadly (see [142] for a *meta*-analysis). The *meta*-analysis by Di Martino and colleagues reported greater likelihood of hypoactivation in ACCg, ACCs and right AI [142], which supports studies of atypical responses to vicarious experience in ASD.

To summarise, vicarious experience may be atypical in individuals with psychopathy and ASD. However, many studies show that these disorders are related to problems with different aspects of empathy (e.g. [9], [111], [112], [119], [132]. Existing work has examined neural responses to the perception of others’ pain or emotional expressions, but not to others reward. In autism the picture of vicarious processing is somewhat mixed, with reduced [139] similar [77], [138] and increased [137] brain responses to others’ pain in AI, ACCg and ACCs.

## 7 Future directions

### 7.1 Implications of research on vicarious experience for psychopathy and ASD

Future studies may benefit from investigating neural responses to others’ reward to examine whether atypical vicarious responses are specific to the processing of distress in others or are reflective of more general insensitivity to social information. Moreover, rewards for others are robustly shown to activate ACCg [26], [51], [52], [53], [55] and there is already evidence that the ACCg may be anatomically and functionally atypical in individuals with ASD and in individuals with psychopathy. For example, Delmonte et al. [143] showed hyperconnectivity between the caudate and ACCg in children with ASD, the strength of which was negatively correlated with neural responses to social rewards [143]. In psychopathy, studies have found grey matter volume and activity in the ACCg to correlate with psychopathic and callous traits [7], [125], [144], [145].

However, many studies have found that these disorders are associated with *different* empathy impairments and the two groups are clearly very different behaviourally [9], [111], [112], [119], [132]. Consequently, whilst ACCg and AI response may be atypical in both disorders this could happen for different reasons, and this is a clear avenue for future research. Moreover, the high comorbidity of autism with alexithymia may help to account for some of the mixed findings [146] as does differences in the specific anatomy of where atypical vicarious activations are observed. Understanding the differences in cognitive and affective components of empathy in psychopathy, ASD, and alexithymia, will also be important in future studies. In particular there is evidence that alexithymia may account for affective empathy and emotion impairments in ASD where they are observed [146], but does not seem to account for cognitive empathy impairments in ASD [119]. Moreover, in the typical population, the reduced affective empathy associated with alexithymia is apparent over and above the reduced affective empathy observed in psychopathy [119]. Structural neuroimaging studies have also shown different neural regions implicated in ASC and alexithymia [147]. Further studies in clinical samples of these disorders could shed light on the nature of these different disorders and the structure of empathy.

### 7.2 Linking vicarious experience to behaviour

Preliminary evidence suggests that individual differences in vicarious experience are linked to variability in behavioural outcomes, such as prosocial and antisocial behaviour, moral judgments and social decision-making (a discussion of which was beyond the scope of this review but see [78], [148]). Moving forward, the application of computational models could help shed light on *how* vicarious information is processed in the brain and is linked to behavioural outcomes, not just where this information my be processed [149]. In particular, the framework of reinforcement learning theory, where learning is driven by prediction errors that signal the difference between expected and actual outcomes of a choice [150] could be used to examine how individual differences in empathy relate to individual differences in social behaviour.

Prediction errors quantify the unexpectedness of outcomes and act as a key learning signal to update future behaviour. This framework has been extensively used to characterise reward-guided behaviour and decision-making. More recently these learning signals, have been identified in social contexts such as during observational learning [151], teaching [53], learning about others’ faces [152] and when interacting with in-group and out-group members [78]. Further studies examining ‘social prediction errors’, prediction errors that result when processing information that is for or about other people, can help us to understand more about the computational functions of different neural regions in social behaviour.

Using computational frameworks could also help to uncover the precise roles of the ACCg, ACCs and AI in vicarious experience and to understand not only how they are involved, but also how they differ. For example, recent theories of the ACCg in social behaviour suggest that this region is encoding the motivation of another person [54], [95]. Recent theories of the computational properties of the AIalso suggest encoding of prediction error signals but in the context of an interoceptive signal about the state of the body (for reviews see [153], [154]). The interplay of these potentially different computational signals could therefore be crucial in vicarious experience.

The use of comparative studies and borrowing well-characterised theories and models of reward-guided behaviour, perception and decision-making, which can be studied across species [155], will also be crucial in future research. Importantly, these studies could consider the specific anatomy of neuroimaging activations. This approach can allow parallels to be drawn between different studies more closely, and perhaps help to infer the functions of particular brain regions [27].

### 7.3 Concluding remarks

Empathy can be defined as the ability to vicariously experience and to understand the affect of other people and is a fundamental aspect of social cognition. Many studies have examined the neural basis of vicarious pain, which has been interpreted as a proxy measure of empathy. More recently research has begun to examine neural responses to others’ reward. Together, these studies have identified regions, such as the ACCg and AI that may be essential for processing information about others. In general, research into vicarious experience in psychopathy and autism has suggested that such experience is atypical. Understanding the functional anatomy of where vicarious information is processed holds promise to provide new insights into how empathy is processed in the brain and behavioural outcomes.
